# MicroRNA-191 regulates oral squamous cell carcinoma cells growth by targeting PLCD1 via the Wnt/β-catenin signaling pathway

**DOI:** 10.1186/s12885-023-11113-9

**Published:** 2023-07-17

**Authors:** Zekun Wang, Wenzhao Guan, Yufeng Ma, Xuedong Zhou, Guohua Song, Jianing Wei, Chenyang Wang

**Affiliations:** 1 State Key Laboratory of Oral Diseases, National Clinical Research Center for Oral Diseases, Department of Cariology and Endodontic Diseases, West China Hospital of Stomatology, Sichuan University, Chengdu, 610041 China; 2 Department of Stomatology, The Second Hospital of Shanxi Medical University, Taiyuan, China; 3 Laboratory Animal Center, Shanxi Key Laboratory of Experimental Animal Science and Human Disease Animal Model, Shanxi Medical University, Taiyuan, 030001 China; 4 Department of Cardiology, Shanxi Provincial Key Laboratory of Cardiovascular Disease Diagnosis, Treatment and Clinical Pharmacology, The Second Hospital of Shanxi Medical University, Taiyuan, China

**Keywords:** Oral squamous cell carcinoma, miR-191, PLCD1, Cell proliferation, Cancer growth and metastasis

## Abstract

**Background:**

Studies have shown that microRNA-191 (miR-191) is involved in the development and progression of a variety of tumors. However, the function and mechanism of miR-191 in oral squamous cell carcinoma (OSCC) have not been clarified.

**Methods:**

The expression level of miR-191 in tumor tissues of patients with primary OSCC and OSCC cell lines were detected using real-time quantitative polymerase chain reaction (RT-qPCR) and western blot. OSCC cells were treated with miR-191 enhancers and inhibitors to investigate the effects of elevated or decreased miR-191 expression on OSCC cells proliferation, migration, cell cycle, and tumorigenesis. The target gene of miR-191 in OSCC cells were analyzed by dual-Luciferase assay, and the downstream signaling pathway of the target genes was detected using western blot assay.

**Results:**

The expression of miR-191 was significantly upregulated in OSCC tissues and cell lines. Upregulation of miR-191 promoted proliferation, migration, invasion, and cell cycle progression of OSCC cells, as well as tumor growth in nude mice. Meanwhile, reduced expression of miR-191 inhibited these processes. Phospholipase C delta1 (PLCD1) expression was significantly downregulated, and negatively correlated with the expression of miR-191 in OSCC tissues. Dual-Luciferase assays showed that miR-191-5p could bind to PLCD1 mRNA and regulate PLCD1 protein expression. Western blot assay showed that the miR-191 regulated the expression of β-catenin and its downstream gene through targeting PLCD1.

**Conclusion:**

MicroRNA-191 regulates oral squamous cell carcinoma cells growth by targeting PLCD1 via the Wnt/β-catenin signaling pathway. Thus, miR-191 may serve as a potential target for the treatment of OSCC.

**Supplementary Information:**

The online version contains supplementary material available at 10.1186/s12885-023-11113-9.

## Background

Oral squamous cell carcinoma (OSCC), the sixth mostcommon malignant tumor in humans, accounts for approximately 2% of all malignancies. 90% of oral carcinoma are squamous cells. There are approximately 350,000 new cases of oral carcinoma and up to 170,000 deaths annually worldwide [1–3]. Although treatments for cancer such as surgery and chemoradiotherapy have improved greatly, the five-year survival rate of patients is still less than 70%, and it is difficult to achieve the purpose of a surgery alone or surgery with adjuvant radiotherapy [4]. Therefore, it is of great significance for the treatment and prognosis to find potential molecular markers and therapeutic targets in the pathogenesis of OSCC.

MicroRNAs (miRNAs) are a class of small non-coding RNAs, which can affect the function of mRNA to regulate gene translation and protein synthesis by complementary pairing with mRNA. Recent studies have reported that multiple miRNAs play important roles in the occurrence and development of tumors by affecting the proliferation, invasion, and migration of tumor cells [5, 6]. Studies have shown that miR-191 is a type of microRNA that plays a role in the occurrence and development of a variety of tumors through different pathways. In follicular thyroid carcinoma, decreased expression of miR-191 could increase tumor growth and cell migration by targeting CDK6 [7]. Whereas in breast cancer, the expression of miR-191 increased, and increased expression of miR-191 promotes the proliferation and migration of breast cancer cells by targeting SATB1 [8]. The expression of miR-191 was also increased in colorectal cancer, and the overexpression of miR-191 promoted the development of human colorectal cancer by targeting C/EBPβ [9]. Studies have also suggested that overexpression miR-191 was involved in angiogenesis [10]. These findings suggest that miR-191 in different tissues can produce multiple biological effects through different mechanisms. However, the function and mechanism of miR-191 in OSCC are remains unclear.

This study aimed to detect the expression of miR-191 in OSCC tissues and cell lines, and to explore the correlation between miR-191 and its target gene as well as its downstream signaling, and clarify the role and mechanism of miR-191 in OSCC. This study may provide new evidence for miR-191 as a promising gene therapy target for OSCC.

## Materials and methods

### Human tissue samples

Samples of OSCC tissues and the adjacent noncancerous tissues (2 cm adjacent to the cancerous tissues) used in this study were obtained from patients with primary OSCC (total of 30 cases) who were hospitalized in the Second Hospital of Shanxi Medical University underwent surgical resection. All patients did not receive chemotherapy or preoperative radiotherapy before surgery. The clinicopathological characteristics of these OSCC tissues are shown in Table 1. This study was approved by the Ethics Committee of the Second Hospital of Shanxi Medical University (2022YX-219), and informed consent was obtained from all patients.

**Table 1:** Clinicopathological characteristics of patients with OSCC

Characteristics	Number of patients （%）
Gender	
Male	18 (60)
Female	12 (40)
Age	
< 60	16 (53.3)
> 60	14 (46.7)
T stage	
T1/T2	27 (90)
T3/T4	3 (10)
TNM stage	
I +II	22 (73.3)
III+ V	8 (26.7)
Site of primary tumor	
Tongue	7 (23.3)
Floor of mouth	1 (3.3)
Buccal mucosa	1 (3.3)
Gingiva	10 (33.3)
Lip	5 (17.7)
Jaw	6 (20.0)
Histologic differentiation	
Well	11 (36.7)
Moderately	16 (53.3)
Poorly	3 (10)
Tumor size	
≤ 4 cm	27 (90)
> 4 cm	3 (10)
Lymph node metastasis	
Positive	5 ( 16.7)
Negative	25 (83.3)

### Cell culture

Human OSCC cells (SCC-9 and CAL-27), and Human normal oral keratinocyte line (hHOK) cells used in this study were purchased from GuanDao Biological Engineering Co., Ltd. (Shanghai, China), human OSCC cells (SCC-4, HSC3, and SAS cells) were purchased from BeNa Bio Inc (Heibei, China). All cells were cultured in high-glucose DMEM medium containing 10% fetal bovine serum (FBS, Gibco, USA) and 1% (v/v) penicillin–streptomycin (Gibco, USA). The cells were incubated at 37 ℃ in a constant temperature incubator with 5% CO_2_ concentration until logarithmic phase.

### RNA extraction and real-time quantitative PCR (RT-qPCR)

Total RNA was extracted using Trizol reagent (Invitrogen, CA, USA). MiRNA, and mRNA were reverse transcribed into respective cDNAs using Mir-X miRNA First-Strand Synthesis and PrimeScript™ RT Reagent Kit (Takara Bio USA, Inc.), and then quantified using TB Green® Premix Ex Taq™ II (Takara Bio USA, Inc.) following the manufacturer’s instructions on a Quant Studio Real-Time PCR system (Applied Biosystems, CA). The expression levels of miR-191 relative to U6, and target genes of PLCD1 relative to GAPDH were determined as respective comparative cycle thresholds (ΔCt). Specific primers used for PLCD1 and GAPDH were as follows: PLCD1 forward 5’-ATGGTGGGACACGGAGTTTG-3’ and reverse 5’-GAGGTGGACATGGCGGTATC-3’; GAPDH forward 5’-GCACCGTCAAGGCTGAG AAC-3’ and reverse 5’-TGGTGAAGACGCCAGTGGA-3’.

### Cell transfection

Following the instructions of transfection reagent Lipofectamine 2000 (Invitrogen, CA, USA), CAL-27 and SCC-9 cells were transfected with miR-191 mimics or miR-191 inhibitor (mimics-NC, and inhibitor-NC was used as a negative control) to increase or inhibit the activity of miR-191. All transfection plasmids were purchased from GenePharma Co., Ltd. (Shanghai, China).

### Detection of cell proliferation

After transfection, cells of each group were seeded into 96 well plates at a density of 4 × 10^3^/well. Cells were supplemented with 10 μL Cell Counting Kit 8 (CCK-8, Boster, Wuhan, China) in each well at 24, 48, 72, and 96 h after seeding, followed by incubation at 37 ℃ for 1 h, and the absorbance value was measured at 450 nm with a microplate reader.

### Detection of cell invasion and migration

Transwell assay was employed to detect cell invasion and migration according to the manufacturer's protocol (BD Biosciences, Bedford, MA, USA). After 24 h of transfection, cells were resuspended in FBS-free DMEM, 100 μL of DMEM medium containing cells (5×10^4^ for SCC-9,1×10^5^ for CAL-27) were added to the upper chamber, and supplemented with 600 μL DMEM medium containing 10% FBS in the lower chamber. After 24 h of incubation, cells were fixed with methanol for 30 min and were stained with 0.1% crystal violet (Boster, Wuhan, China). Five fields were randomly selected for cell counting under microscope.

### Detection of cell cycle

Forty-eight hours after transfection, cells were fixed with 75% ethanol for 24 h. After discarding ethanol, cells were resuspended in PBS and then stained using a cell cycle detection kit (Invitrogen, CA, USA) for 30 min, after which the cell cycle was measured by flow cytometry.

### Western blot assay

Total protein was obtained using a protein extraction reagent (ThermoFisher, USA). The proteins were separated by sodium dodecylsulphate polyacrylamide gel electrophoresis (SDS-PAGE) and transferred to a polyvinylidene fluoride (PVDF) membrane (Millipore, USA). PVDF membranes were then blocked using 5% bovine serum albumin and placed at 4 ℃ overnight after the addition of primary antibodies. PVDF membranes were subsequently incubated in Horseradish Peroxidase (HRP) conjugated secondary antibodies (BIO-RAD, USA) for 1 h at room temperature. ECL chromogenic kit (Boster, Wuhan, China) was used for chromogenic development, and the gray values of the bands were analyzed.

### Prediction and identification of target genes

#### Dual Luciferase assay

The target genes of miR-191 were predicted by bioinformatics software. The target gene of miR-191 was predicted by three software (MiRanda, TargetScan, and PicTar) and the human PLCD1 3'-UTR region targeted by miR-191 was amplified by PCR, and cloned into the PLCD1-3'-UTR plasmid obtained from luciferase assay to construct PLCD1-WT. Plasmid PLCD1-MUT with mutant binding site was also constructed as control. OSCC cells were co-transfected with pmiR-PLCD1-WT / pmiR-PLCD1-MUT, and miR-191 mimics or negative controls by using Lipofectamine 2000. Forty-eight hours after transfection, luciferase activity was measured using dual- luciferase reporter assay system according to the instructions of manufacturer.

#### Regulation of PLCD1 expression by miR-191

To further confirm the regulation of miR-191 on PLCD1 expression, RT-qPCR and western blot assays were used to detect the expression of PLCD1 in OSCC tissues and OSCC cells. MiR-191 mimics, miR-191 inhibitor, and NC were transfected into OSCC cells, and the mRNA expression level of PLCD1 in cells were detected and compared.

#### Analysis of the effect of miR-191 on proliferation, invasion and migration of OSCC cells by regulating expression of PLCD1

To further verify whether miR-191 inhibitor affect the proliferation, migration and invasion of OSCC cells by upregulating PLCD1, the OSCC cells were co-transfected with miR-191 inhibitor +small interfering RNA (siRNA) -NC, and miR-191 + siRNA-PLCD1. At 24, 48, 72, and 96 h after transfection, MTT assay was used to detect cell proliferation. Transwell assay was used to detect cell invasion and migration at 48 h after transfection.

#### Determination of the effect of miR-191 on the PLCD1 downstream signaling pathway

In order to further clarify the mechanism of miR-191 regulating OSCC cells growth, the protein expression of β-catenin in PLCD1 downstream signaling pathway was detected in OSCC cells transfected with miR-191 mimics / miR-191 inhibitor. The downstream genes expression of the β-catenin signaling pathway, including C-myc, matrix metalloproteinase-9 (MMP-9), CDK4 and PCNA protein were also detected. Since β-catenin pathway is involved in epithelial-mesenchymal transition (EMT) process, the EMT-related marker N-cadherin was further detected. The siRNA-PLCD1 was used to inhibit mRNA of PLCD1 to verify the effect of miR-191 on β-catenin, MMP-9, C-myc, N-cadherin, CDK4 and PCNA expression through PLCD1.

### Xenograft model

Animal experiments in this study were approved by the Ethics Committee of the Second Hospital of Shanxi Medical University (DW2022069) and were performed in accordance with Guide for the Care and Use of Laboratory Animals published by National Institutes of Health. Healthy female BALB/c nude mice (4 weeks old) were purchased from Gempharmatech Co., Ltd. (Jiangsu, China). According to the instruction of Lipofectamine 2000 reagent, OSCC cells were transfected with miR-191 enhancer (miR-191 agomir) and negative control (miR-191 agomir NC), miR-191 inhibitor (miR-191 antagomir), and negative control (miR-191 antagomir NC). At 48 h after cell transfection, 0.2 mL of OSCC cell suspension containing 2 × 10^6^ cells was injected subcutaneously into the right axillary region of nude mice. Treatment was started on 7 day after cell injection. MiR-191 agomir, miR-191 antagomir and negative controls were injected directly into the tumors every 7 days. Tumor size was monitored by measuring tumor length (L) and width (W) with vernier calipers every 7 days prior to subsequent injection, and tumor volume was calculated using the following formula: volume (mm^3^) = length × width^2^× 0.5. After 28 days, the nude mice were sacrificed. The tumors in vivo were removed, weighed, and photographed.

### Data analysis

All statistical analyses and plots were performed and generated using GraphPad Prism software (version 8.0; GraphPad Software, Inc, CA, USA). Student’s T-test and One-way analysis of variance (ANOVA) were used to compare the difference between groups. Data correlation between the two groups were analyzed by Pearson correlation. The measurement data were presented as the mean ± standard deviation (SD), and a *P*-value less than 0.05 or 0.01 was considered statistically significant.

## Results

### Increased expression of miR-191 in OSCC tissues and cell lines

**Fig. 1:**
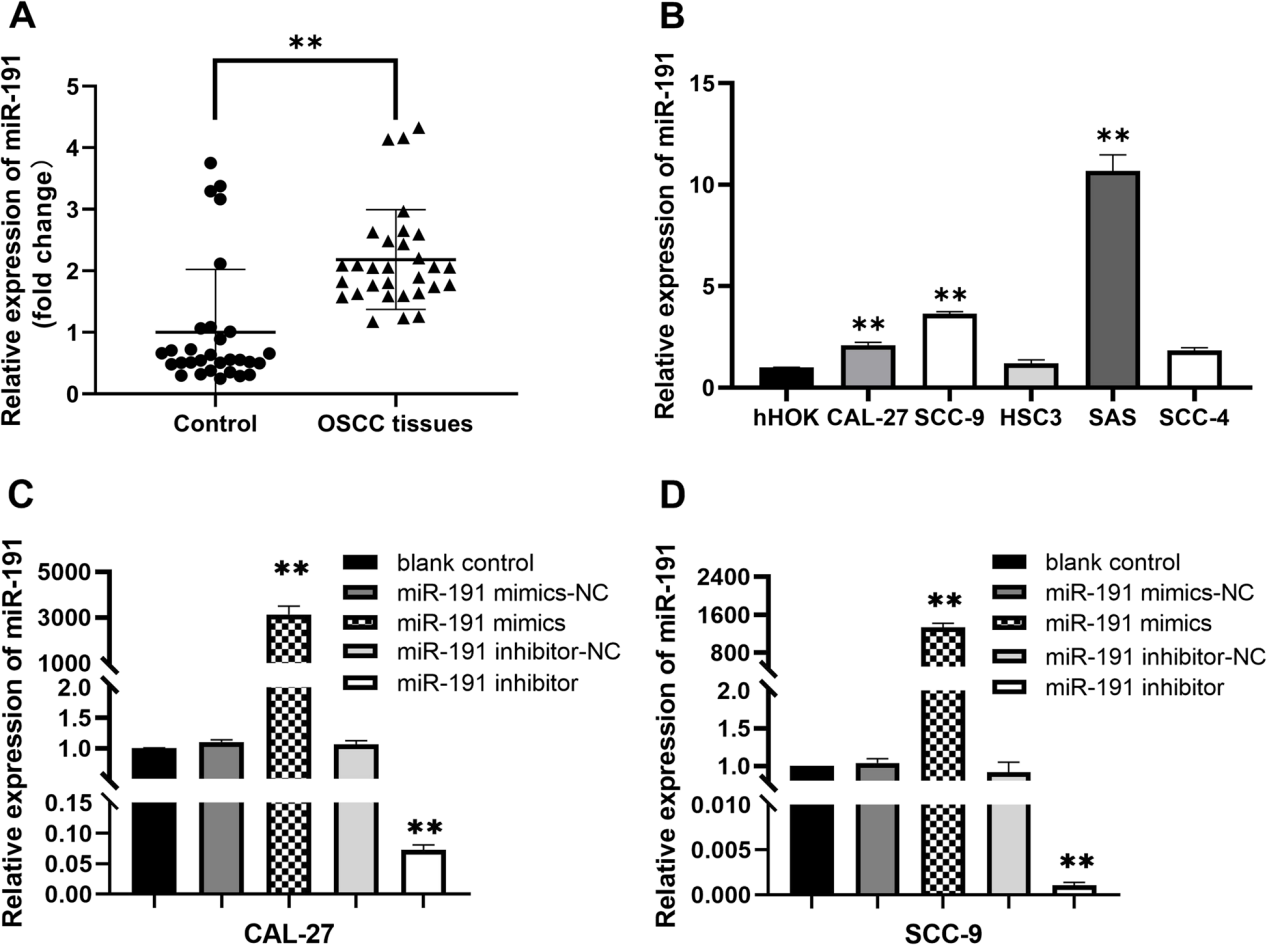
The expression level of miR-191 in OSCC tissues and cell lines. **A** The expression of miR-191 in 30 OSCC tissues and normal noncancerous tissues were detected using RT-qPCR assay (*n* = 30). **P < 0.01 vs. normal tissues. **B** The expression of miR-191 in CAL-27, SCC-9, SAS, SCC-4, HSC3 and HOK cells were detected using RT-qPCR assay (*n* = 3). **P < 0.01 vs. HOK cells. **C**, **D** The miR-191 mimics transfection significantly increased the expression of miR-191 and the miR-191 inhibitor transfection significantly decreased the expression of miR-191 in CAL-27 and SCC-9 cells (*n* = 3); Data represented as means ± SD. **P < 0.01 vs. mimics / inhibitor negative control. Blank control: without any transfection

The expression level of miR-191 in OSCC tissues and cell lines were detected by RT-qPCR. As shown in Fig. 1A, the results demonstrated that significant upregulation of miR-191 expression was observed in OSCC tissue samples from patients compared with controls (adjacent noncancerous tissues). Compared with hHOK cells, the expression of miR-191 in CAL-27, SCC-9, and SAS cells were significantly increased, there was no statistical difference between the SCC-4 and HSC3 cells. Hence, CAL-27 and SCC-9 cells were chosen for further investigation in this study (Fig. 1B).

### Effects of miR-191 mimics or inhibitor transfection on proliferation, migration, invasion, and cell cycle in CAL-27, and SCC-9 cells

CAL-27, and SCC-9 cells were transfected with miR-191 mimics or miR-191 inhibitor, respectively. After 48 h of transfection, RT-qPCR assay showed that miR-191 expression was significantly increased in cells from the miR-191 mimics group compared with cells from the miR-191 mimics-NC group, and decreased in cells from the miR-191 inhibitor group compared with cells from the miR-191 inhibitor-NC group, there was no significant difference between the blank group and the inhibitor NC groups (Fig. 1C, D).

**Fig. 2:**
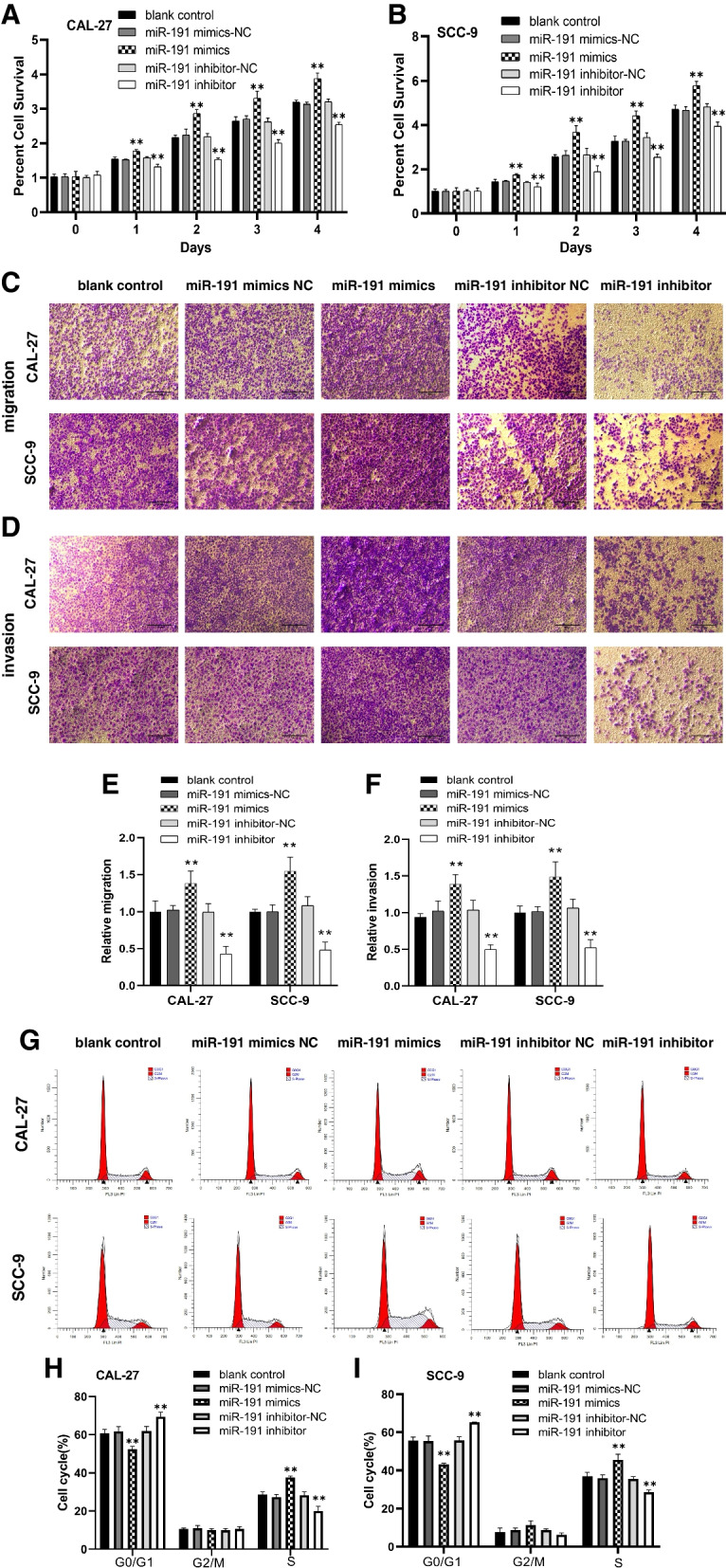
Effects of miR-191 mimics or inhibitor transfection on proliferation, migration, invasion and cell cycle in CAL-27 and SCC-9 cells. **A**, **B** CAL-27 and SCC-9 cells proliferation in the miR-191 mimics group and miR-191 inhibitor group detected using MTT. The percent cell survival and was normalized to that of control cells (*n* = 3). **p < 0.01. **C**, **D** The representative images of cell migration and invasion detected using transwell chambers (scale bar, 100 μm). **E**, **F** The migration and invasion rates in miR-191 mimics group and miR-191 inhibitor group (*n* = 3). **p < 0.01 vs. mimics / inhibitor negative control group. **G** Representative images of cell cycle distribution which was detected using flow cytometer. **H**, **I** The percentage of cells in G0/G1 phase, and S phase in CAL-27, and SCC-9 cells transfected with miR-191 mimics and miR-191 inhibitor (*n* = 3). **p < 0.01 vs. mimics / inhibitor negative control group

CCK-8 assay and Transwell assay showed that cell proliferation, cell migration and invasion were significantly increased in the miR-191 mimics group compared with the cells in the miR-191 mimics-NC group and decreased in the miR-191 inhibitor group compared with the cells in the miR-191 inhibitor-NC group (Fig. 2A-F).

Flow cytometry assay showed that transfection with miR-191 mimics promoted cell cycle progression in CAL-27, and SCC-9 cells, resulting in a significant decrease in the percentage of cells in G1 phase and a parallel increase in the percentage of cells in S phase, whereas transfection with miR-191 inhibitor induced cell cycle arrest in CAL-27, and SCC-9 cells, resulting in an increase in the number of cells in G1 phase and a decrease in the number of cells in S phase (Fig. 2G-I).

### MiR-191 can bind to the 3′-UTR of PLCD1

**Fig. 3:**
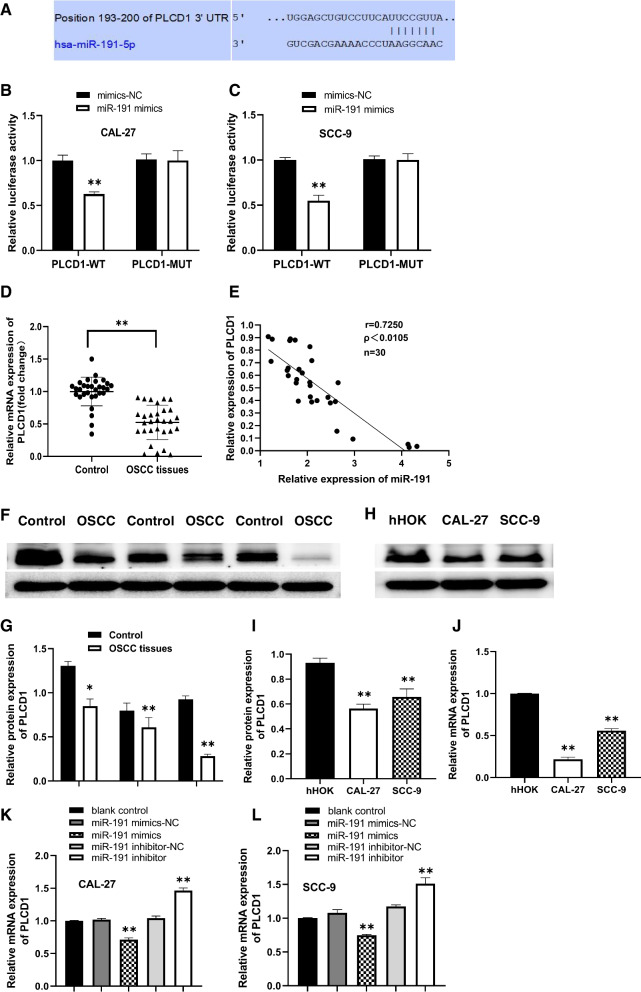
PLCD1 is a direct target gene of miR-191, and miR-191 regulates the expression of PLCD1. **A** Schematic illustration of the position of miR-191 target site in 3′-UTR of PLCD1 mRNA. **B**, **C** Effect of miR-191 on Luciferase activity of PLCD1-WT and PLCD1-MUT in OSCC cells (*n* = 3). **p < 0.01 vs. mimics-NC; **D** The mRNA expression of PLCD1 in OSCC tissues (*n* = 30). **p < 0.01 vs. normal tissues; **E** The mRNA expression of PLCD1 in OSCC tissues was negatively correlated with the expression of miR-191 in OSCC tissues. **F** The PLCD1 protein expression in OSCC tissues. **G** Comparison of the PLCD1 protein in OSCC tissues with adjacent normal tissues (*n* = 3). *p < 0.05 and **p < 0.01 vs. normal tissues; **H** The PLCD1 protein expression in CAL-27, and SCC-9 cells detected using western blot assay. **I** Comparison of the PLCD1 protein expression in CAL-27, SCC-9, and HOK cells (*n* = 3). **p < 0.01 vs. HOK cells; **J** Comparison of the PLCD1 mRNA expression in CAL-27, SCC-9, and HOK cells (*n* = 3). **p < 0.01 vs. HOK cells; **K**, **L** The mRNA expression of PLCD1 in CAL-27 and SCC-9 cells transfected with miR-191 mimics and miR-191 inhibitor (*n* = 3). **p < 0.01 vs. mimics / inhibitor negative control group. We cropped the blots before antibody hybridization. The full-length membranes, with membrane edges visible, are presented in Supplementary Fig. 1

The results of luciferase assays showed that transfection of miR-191 mimics significantly reduced the luciferase activity of PLCD1-WT in OSCC cells, but the luciferase activity of PLCD1-MUT in OSCC cells did not change significantly. These data indicated that PLCD1 was one of the direct targets of miR-191 (Fig. 3A-C).

### Correlation between miR-191 and PLCD1 in OSCC tissues and cells

#### The opposite expression level of miR-191 and PLCD1 in OSCC tissues 

Results of RT-qPCR assay showed that the expression level of PLCD1 mRNA in OSCC tissues of 30 OSCC patients were significantly downregulated compared with adjacent noncancerous tissues (Fig. 3D). The results of correlation analysis assay showed that the expression level of PLCD1 mRNA in OSCC tissues was negatively correlated with the expression of miR-191 in OSCC tissues (Fig. 3E).

#### Regulatory effect of miR-191 on PLCD1

Western blot assay showed that PLCD1 protein expression was significantly downregulated in OSCC tissues compared with adjacent noncancerous tissues (Fig. 3F-G), and the PLCD1 mRNA and protein expression were significantly downregulated in CAL-27 and SCC-9 cells compared with hHOK cells (Fig. 3H-J).

There is a significant decrease in PLCD1 mRNA expression of CAL-27 and SCC-9 cells transfected with miR-191 mimics compared with miR-191 mimics negative control and blank control. In contrast, PLCD1 mRNA level was significantly upregulated in CAL-27 and SCC-9 cells transfected with miR-191 inhibitor compared with miR-191 inhibitor negative control and blank control (Fig. 3K-L).

#### Effect of PLCD1 inhibition on the downregulation of miR-191 on the proliferation, invasion and migration of OSCC cells

**Fig. 4:**
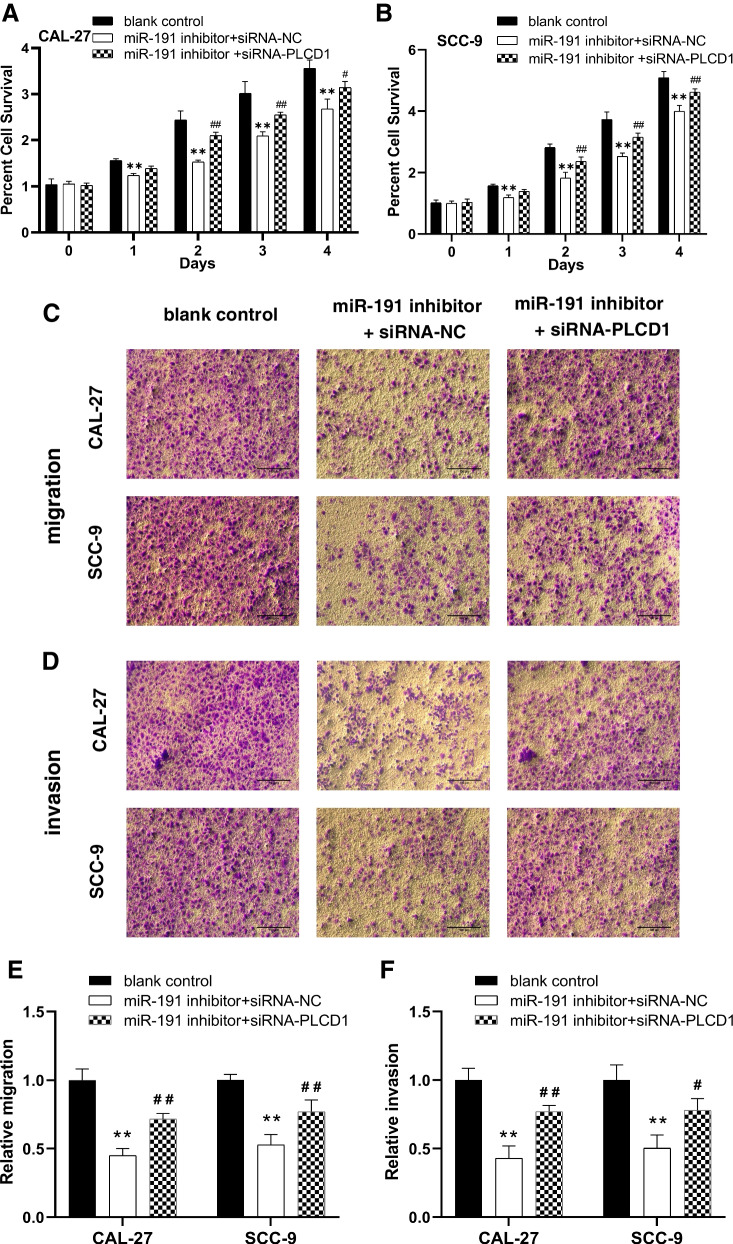
The effect of PLCD1 inhibition on the downregulation of miR-191 on the proliferation, migration and invasion of OSCC cells. **A**, **B** The proliferation ability of CAL-27 and SCC-9 cells after downregulating miR-191 and downregulating PLCD1 detected using MTT. The percent cell survival and was normalized to that of control cells (*n* = 3).**p < 0.01. **C**, **D** Representative images of PLCD1 inhibition on the downregulation of miR-191 on the migration and invasion of CAL-27, and SCC-9 cells (scale bar, 100 μm). **E**, **F** Transwell cell migration and invasion assay showing the migration and invasion ability of CAL-27, and SCC-9 cells (*n* = 3). **p < 0.01 vs. blank control, ^#^*p* < 0.05 and ^##^*p* < 0.01 vs. miR-191 inhibitor + si-NC

To further verify whether miR-191 inhibitor play a role in inhibiting OSCC cell proliferation, migration and invasion by upregulating PLCD1, OSCC cells were transfected with siRNA-PLCD1 and siRNA-NC. Results showed that the proliferation, migration and invasion ability of OSCC cells was significantly inhibited after downregulating miR-191, while the proliferation, migration and invasion ability was partially restored by downregulating PLCD1 on the basis of downregulating miR-191 (Fig. 4A-F). These results indicate that miR-191 promoted proliferation, migration and invasion of OSCC cells by negatively regulating PLCD1 expression.

#### MiR-191 regulates the genes expression of PLCD1 downstream signaling pathway

**Fig. 5:**
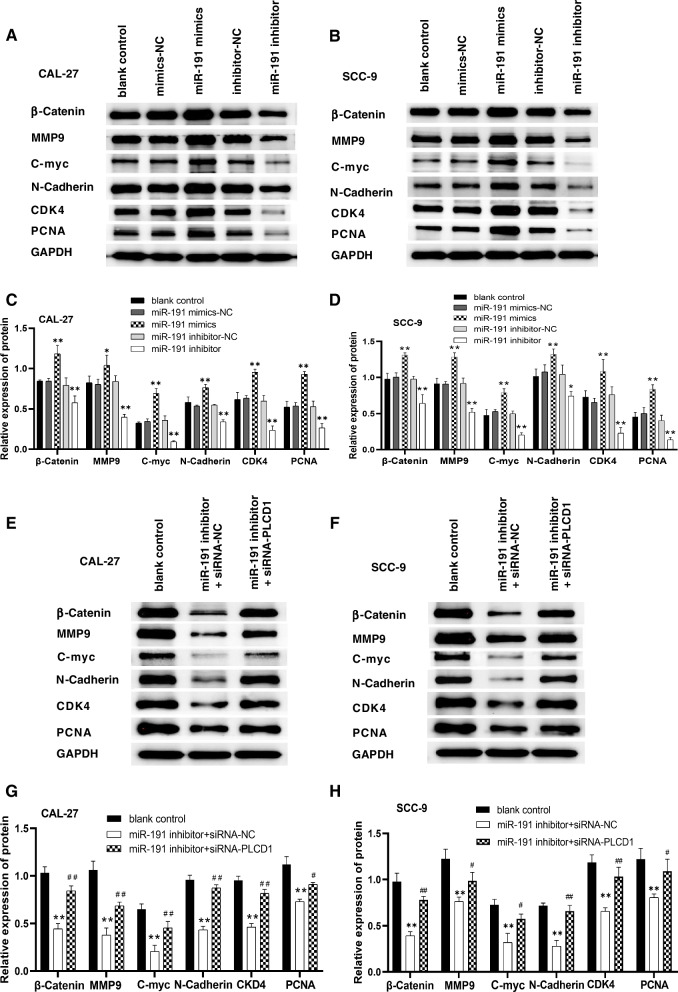
MiR-191 regulates the expression of β-catenin and the downstream genes of the β-catenin signaling pathway through targeting PLCD1. **A**, **B** The protein expression of β-catenin, MMP-9, C-myc, N-cadherin, CDK4 and PCNA in CAL-27, and SCC-9 cells detected using western blot. **C**, **D** Overexpression of miR-191 increased the protein expression of β-catenin, MMP-9, C-myc, N-cadherin, CDK4 and PCNA in CAL-27 and SCC-9 cells, and downregulation of miR-191 inhibited these genes expression (*n* = 3). *p < 0.05 and **p < 0.01 vs. mimics / inhibitor negative control group. **E**, **F** The β-catenin, MMP-9, C-myc, N-cadherin, CDK4 and PCNA protein expression in OSCC cells were detected using western blot assay. **G**, **H** Downregulation of miR-191 inhibited the expression of β-catenin, MMP-9, C-myc, N-cadherin, CDK4 and PCNA in CAL-27 and SCC-9 cells; while downregulation of PLCD1 on the basis of downregulation of miR-191 could partially recover the effect of downregulation of miR-191 on the expression of β-catenin, MMP-9, C-myc, N-cadherin, CDK4 and PCNA in CAL-27 and SCC-9 cells (*n* = 3). **p < 0.01 vs Control group, ^#^*p* < 0.05 and ^##^*p* < 0.01 vs miR-191 inhibitor + siRNA-NC group. We cropped the blots before antibody hybridization. The full-length membranes, with membrane edges visible, are presented in Supplementary Fig. 1

Western blot assay showed that overexpression of miR-191 increased the protein expression of β-catenin, MMP-9, C-myc, N-cadherin, CDK4 and PCNA in OSCC cells. Inhibition of miR-191 decreased the protein expression of β-catenin, MMP-9, C-myc, N-cadherin, CDK4 and PCNA in CAL-27 and SCC-9 cells (Fig. 5A-D).

Western blot assay was further used to verify the effect of downregulation of miR-191 on the expression of β-catenin, MMP-9, C-myc, N-cadherin, CDK4 and PCNA protein in OSCC cells on the basis of downregulation of PLCD1. The results showed that downregulation of miR-191 inhibited the expression of β-catenin, MMP-9, C-myc, N-cadherin, CDK4 and PCNA protein in OSCC cells, and downregulation of PLCD1 on the basis of downregulation of miR-191 could partially recover the effect of downregulation of miR-191 on the expression of β-catenin, MMP-9, C-myc, N-cadherin, CDK4 and PCNA in OSCC cells, suggesting that miR-191 regulates the expression of β-catenin, MMP-9, C-myc, N-cadherin, CDK4 and PCNA through targeting PLCD1 (Fig. 5E-H).

#### MiR-191 expression promotes the growth of OSCC in vivo

**Fig. 6:**
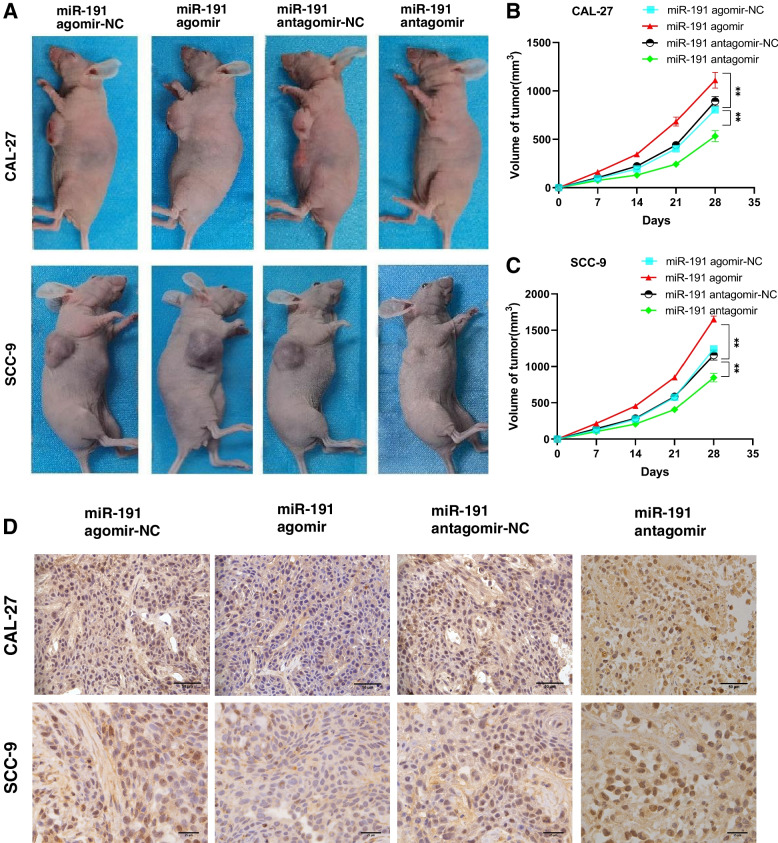
miR-191 overexpression promotes the growth of OSCC in vivo, miR-191 inhibitior reduced tumor volumes in nude mice through targeting PLCD1. **A** Representative photographs of mice and tumors. **B**, **C** Tumor sizes between miR-191 agomir group and miR-191 antagomir group. **D** IHC assay of PLCD1 expression in miR-191 agomir group and miR-191 antagomir group. (scale bar, 25 μm (SCC-9) and 50 μm (CAL-27)). **p < 0.01 vs. miR-191 agomir / miR-191 antagomir negative control group

Compared with miR-191 agomir-NC, miR-191 agomir promoted tumor formation and growth in nude mice, and the tumor size was significantly increased. However, compared with the miR-191 antagomir-NC, miR-191 antagomir inhibited the tumor growth, which resulted in a significant reduction in tumor size in nude mice (Fig. 6A-C). Immunohistochemistry (IHC) assay showed that the PLCD1 expression was significantly downregulated in tumors tissue from the miR-191 agomir group and upregulated in tumors tissue from the miR-191 antagomir group, suggesting that miR-191 promotes OSCC tumor growth in vivo by regulating protein expression of PLCD1 (Fig. 6D).

## Discussion

MiRNAs functions as oncogenes or tumor suppressor genes in many human tumors, affecting various stages of tumor formation and development, which can be used for early diagnosis and prognosis evaluation of tumors [11]. In recent years, several studies have shown that miR-191 is abnormally expressed in a variety of tumor tissues and plays a role in tumor initiation and progression through different mechanisms [12–18]. However, the function and mechanism of miR-191 in OSCC have not been clarified.

This study showed that expression level of miR-191 in OSCC tissues and cell lines was significantly higher than in adjacent non-neoplastic tissues and hHOK. In vitro results showed that miR-191 overexpression promoted cell proliferation, migration, invasion and cell cycle in OSCC cells. In vivo experiments showed that miR-191 overexpression promoted tumorigenesis of OSCC cells in nude mice. In contrast, downregulation of miR-191 expression inhibited proliferation, migration and invasion of OSCC cells, and inhibited tumorigenesis of OSCC cells in nude mice.

PLCD1 encodes an enzyme involved in energy metabolism, calcium homeostasis, and intracellular movement. It is located at 3p22 in a region that is frequently deleted in multiple cancers [19]. Several studies have shown that PLCD1 has anti-cancer effects. PLCD1 has been confirmed to play a cancer suppressive effect in colorectal cancer [20], gastric cancer [21], esophageal squamous carcinoma [22], pancreatic cancer [23], breast cancer [24], chronic myeloid leukemia [25]. Studies have confirmed that overexpression of PLCD1 can promote the apoptosis of colorectal tumor cells, arrest the cell cycle at G1 / S phase, and inhibit proliferation, invasion and migration of colorectal tumor cells [26]. Mu et al. [27] found that PLCD1 could significantly promote G2 / M cell cycle arrest and apoptosis in human breast cancer cells, thereby significantly inhibiting the proliferation of human breast cancer cells. However, the function and mechanism of PLCD1 in OSCC have not been clarified.

In vitro experiments, this study demonstrated that miR-191 could directly target the 3′-UTR of PLCD1 and the expression of miR-191 was negatively correlated with expression of PLCD1. Inhibiting the expression of miR-191 significantly increased the expression of PLCD1 in OSCC cells, and inhibited the proliferation, migration and invasion of OSCC cells, suggesting that miR-191 promoted the proliferation, migration, invasion and cell cycle of OSCC cells by targeting PLCD1. In vivo animal tests further proved these conclusions that overexpressed miR-191 promoted tumor growth and invasion in nude mice by inhibiting PLCD1, and decreasing miR-191 expression promoted PLCD1 and reduced tumor growth and infiltration in nude mice.

Activation of the Wnt/β-catenin pathway has been found in many human tumors [28], and activation of its target genes can promote tumor development by affecting cell migration, proliferation, and angiogenesis [29]. A study from He et al. [19] proved that PLCD1 could downregulate expression of β-catenin, leading to a decrease in the level of phosphorylated β-catenin. Upregulation of PLCD1 can inhibit the activity of β-catenin pathway in esophageal cancer. Our study demonstrated that overexpressed miR-191 promoted expression of β-catenin, and inhibition of miR-191 expression could decrease activity of β-catenin, suggesting that miR-191 could affect the proliferation, migration and invasion of OSCC cells through β-catenin pathway by targeting PLCD1. C-myc has been recognized as a proto-oncogene that can be malignant by uncontrolled cell proliferation due to its overexpression, and the degradation of extracellular matrix by MMP-9 plays a critical role in tumor invasion and metastasis [30, 31]. CDK4 overexpression can activate the progression of the cell cycle, and regulates progression from G1 to S phase of the cell cycle. Dysregulated CDK4 contributes to abnormal cell proliferation and tumor development [32]. Proliferating cell nuclear antigen (PCNA) is closely related to DNA synthesis and cell cycle in cells [33]. Studies have shown that β-catenin signaling pathway can promote proliferation and metastasis of tumor cells by activating the transcription of various oncogenes, such as MMP9, C-myc, and CDK4 [32, 34–36]. Therefore, we examined the effect of enhancing or inhibiting miR-191 on the expression of MMP9, C-myc, and CDK4 which are the major target genes in the downstream pathway of β-catenin. We further examined the effect of enhancing or inhibiting miR-191 on the expression of PCNA. Western blot assay showed that the expression of MMP9, C-myc, CDK4 and PCNA significantly increased after enhancing miR-191 expression, and significantly decreased after inhibiting miR-191 expression.

EMT was closely related to cell migration and invasion ability [37, 38]. Since β-catenin pathway is involved in EMT process [39], we further explored whether miR-191-mediated β-catenin increasing affects EMT-related marker N-cadherin. Western blot results showed that N-cadherin gene expression was significantly increased after overexpression of miR-191, and inhibition of miR-191 significantly decreased the expression of N-cadherin.

In order to verify the effect of downregulation of miR-191 on the expression of β-catenin, MMP-9, C-myc, N-cadherin, CDK4 and PCNA protein in OSCC cells on the basis of downregulation of PLCD1. OSCC cells were co-transfected with siRNA-PLCD1 and miR-191 inhibitor, and the expression of β-catenin, C-myc, MMP9, N-cadherin, CDK4 and PCNA detected by western blot assay. The results showed that downregulation of miR-191 inhibited the expression of β-catenin, MMP-9, C-myc, N-cadherin, CDK4 and PCNA protein in OSCC cells, and downregulation of PLCD1 on the basis of downregulation of miR-191 could partially recover the effect of downregulation of miR-191 on the protein expression of β-catenin, MMP-9, C-myc, N-cadherin, CDK4 and PCNA in OSCC cells, suggesting that miR-191 regulates the expression of β-catenin, MMP-9, C-myc, N-cadherin, CDK4 and PCNA through targeting PLCD1.

These results suggest that miR-191 promotes proliferation, migration and invasion of OSCC cells, which may be associated with the decrease of PLCD1 and then increase of β-catenin, and ultimately lead to the increase of MMP-9, C-myc, N-cadherin, CDK4 and PCNA. Inhibition of miR-191 increase PLCD1, and then decrease β-catenin, MMP-9, C-myc, N-cadherin, CDK4 and PCNA, ultimately leading to the decrease of proliferation, migration and invasion of OSCC cells, thus exerting anti-tumor effects.

## Conclusion

In summary, our study verified that miR-191 plays a role in oral carcinogenesis and development of OSCC through its target gene PLCD1 and downstream β-catenin signaling pathway of PLCD1. Suppression of miR-191 could inhibit OSCC cells proliferation, migration and invasion. These findings suggest that miR-191 may be a therapeutic target for future clinical treatment of OSCC.

## Supplementary Information

**Additional file 1.**

## Data Availability

The datasets used and/or analyzed during the current study are available from the corresponding author on request.

## Abbreviations

OSCC: Oral squamous cell carcinoma
cDNA: Complementary DNA
RT-qPCR: Real-time quantitative polymerase chain reaction
PLCD1: Phospholipase C delta1
miRNAs: MicroRNAs
CCK-8: Cell Counting Kit 8
3'-UTR: 3′-Untranslated regions
WT: Wild type
MUT: Mutant type
MMP-9: Matrix metalloproteinase-9
siRNA: Small interfering RNA
EMT: Epithelial-mesenchymal transition
SDS-PAGE: Sodium dodecylsulphate polyacrylamide gel electrophoresis
PVDF: Polyvinylidene fluoride
HRP: Horseradish Peroxidase
SD: Standard deviation
