# Supplementary material for: MicroRNA-191 regulates oral squamous cell carcinoma cells growth by targeting PLCD1 via the Wnt/β-catenin signaling pathway
Source: BMC Cancer. 2023 Jul 17;23:668. doi: 10.1186/s12885-023-11113-9 (PMC10351167; doi:10.1186/s12885-023-11113-9)

Fig3F: The PLCD1 protein expression in OSCC tissues.

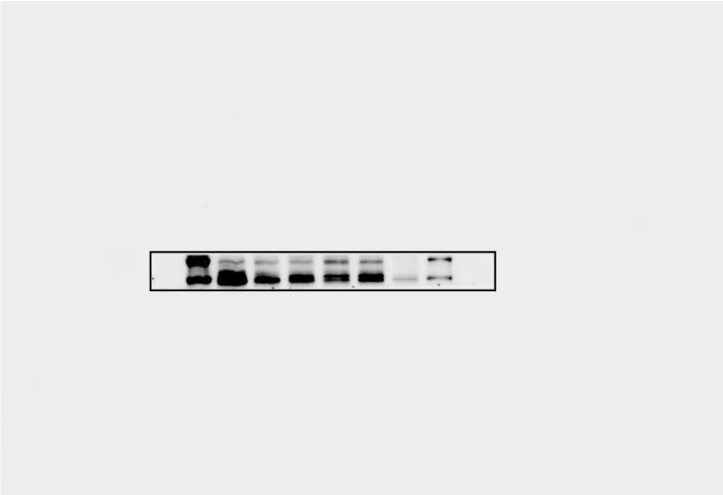

Fig3F: The GAPDH protein expression in OSCC tissues.

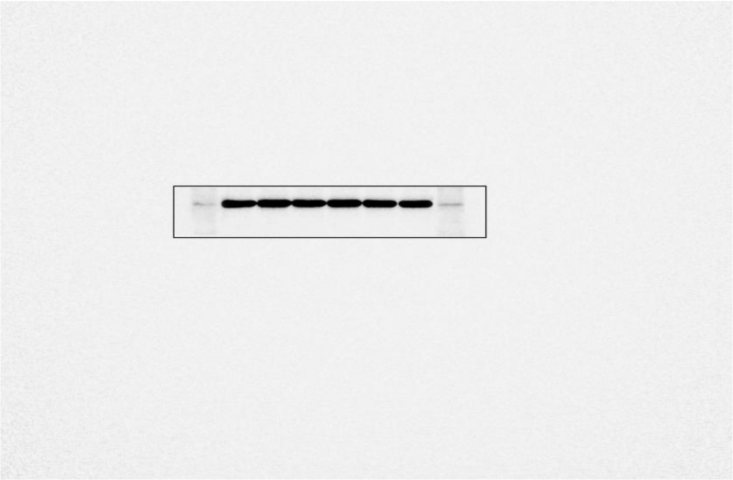

Fig3H: PLCD1 protein expression in CAL-27, SCC-9, and HOK cells.

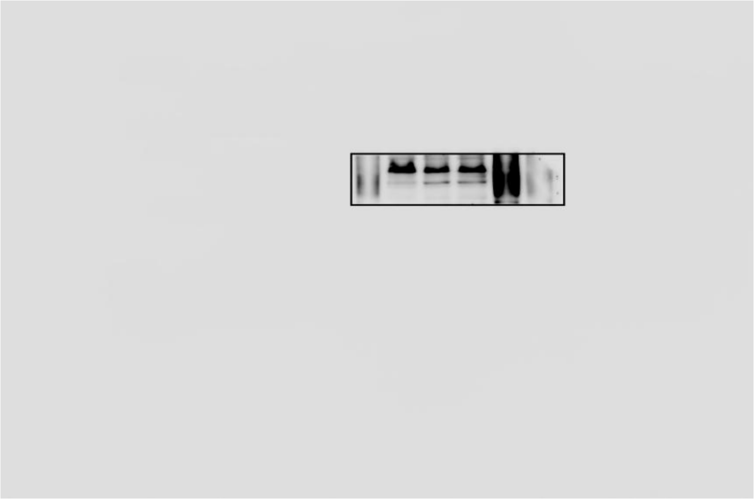

Fig3H: GAPDH protein expression in CAL-27, SCC-9, and HOK cells.

CAL-27 SCC-9 HOK

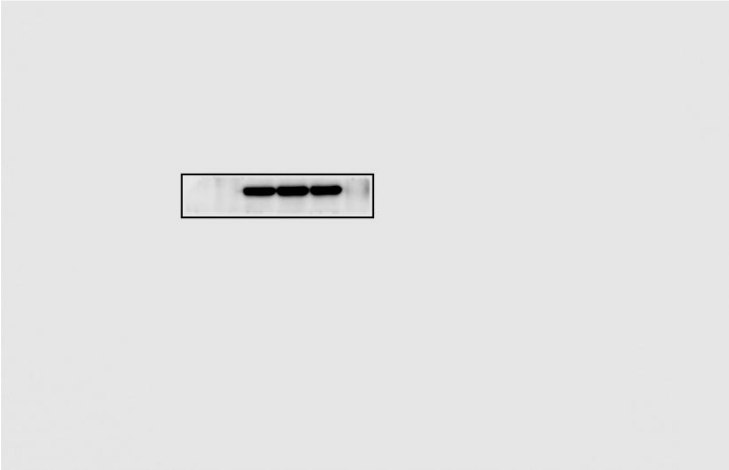

Fig5A: MiR-191 regulates the expression of β-catenin and the downstream genes of the β-catenin signaling pathway. Blank-control(C), miR-191mimics-NC(M-NC), miR-191mimics (M). miR-191inhibitor-NC(I-NC), miR-191 inhibitor (I)

CAL-27- β-catenin C, M-NC, M, I-NC, I

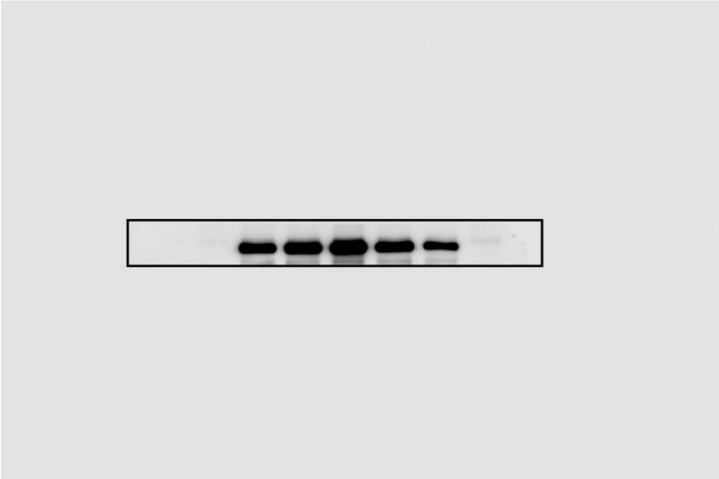

CAL-27-MMP-9 C, M-NC, M, I-NC, I

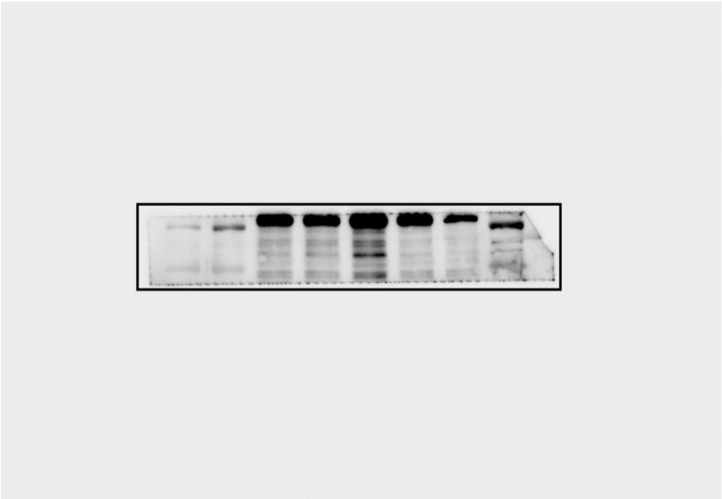

CAL-27-C-myc C, M-NC, M, I-NC, I

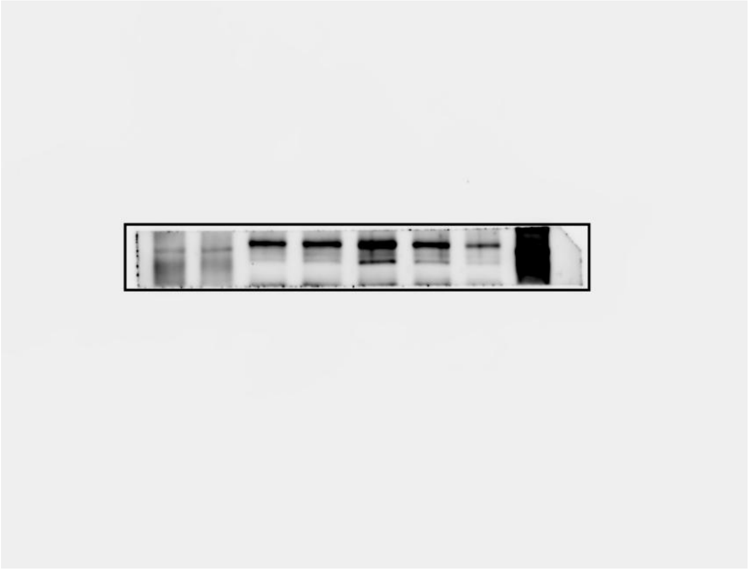

CAL-27--N -Cadherin C, M-NC, M, I-NC, I

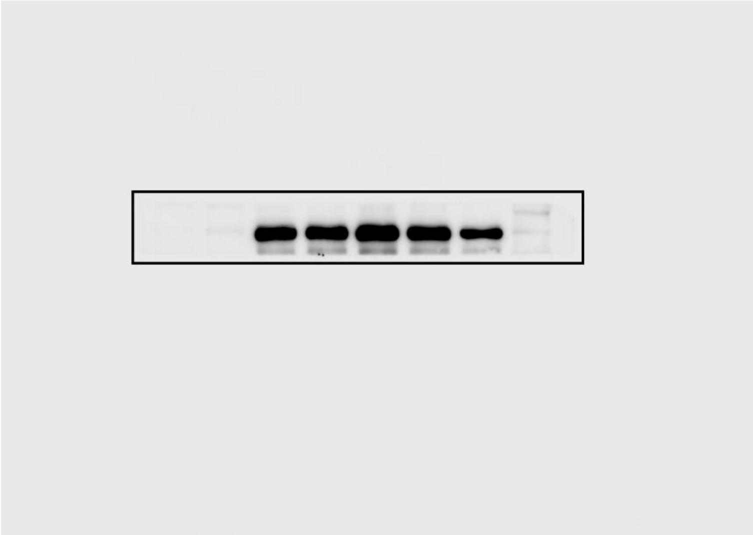

CAL-27-CDK4 C, M-NC, M, I-NC, I

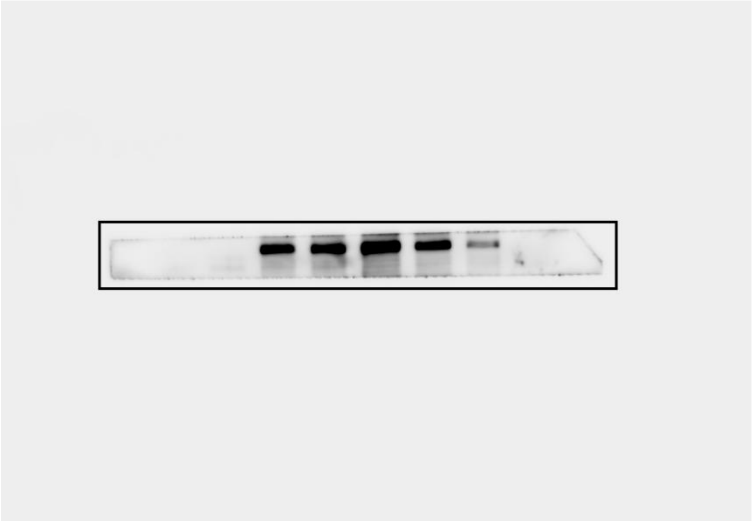

CAL-27-PCNA C, M-NC, M, I-NC, I

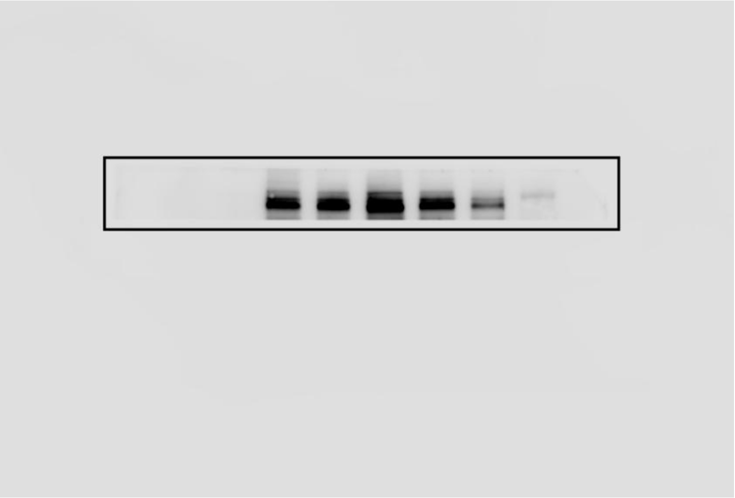

CAL-27-GAPDH C, M-NC, M, I-NC, I

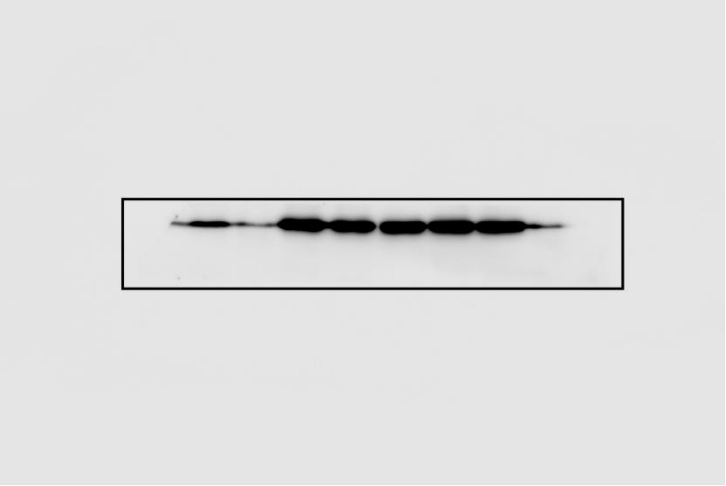

Fig5B: MiR-191 regulates the expression of β-catenin and the downstream genes of the β-catenin signaling pathway.

SCC-9-β-catenin C, M-NC, M, I-NC, I

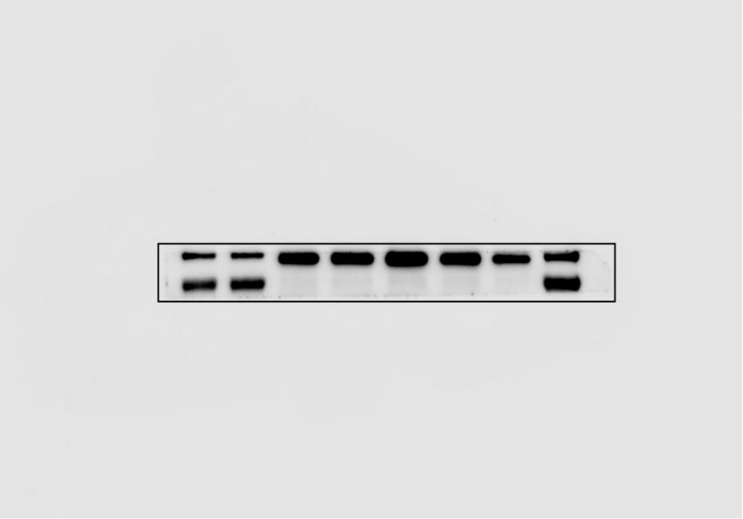

SCC-9-MMP-9 C, M-NC, M, I-NC, I

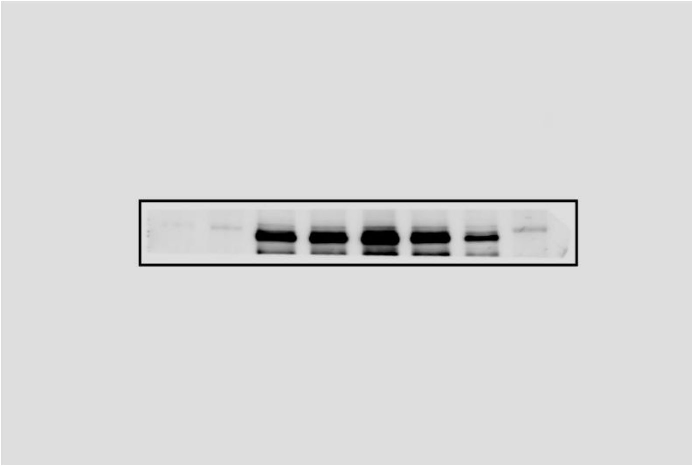

SCC-9-C-myc C, M-NC, M, I-NC, I

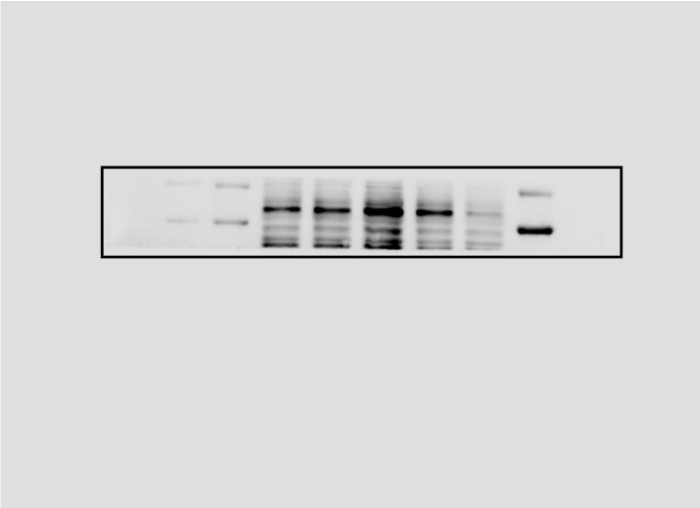

SCC-9-N-cadherin C, M-NC, M, I-NC, I

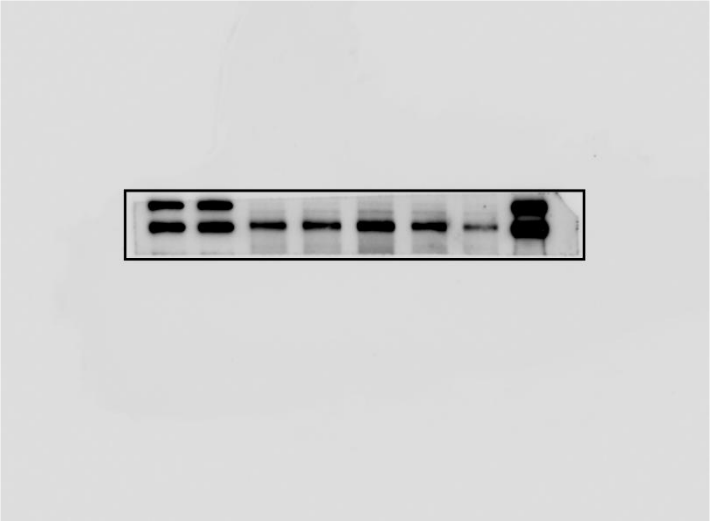

SCC-9- CDK4 C, M-NC, M, I-NC, I

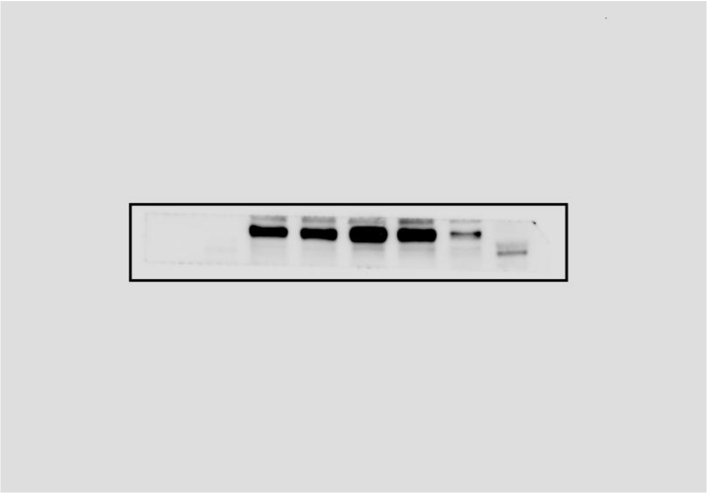

SCC-9-PCNA C, M-NC, M, I-NC, I

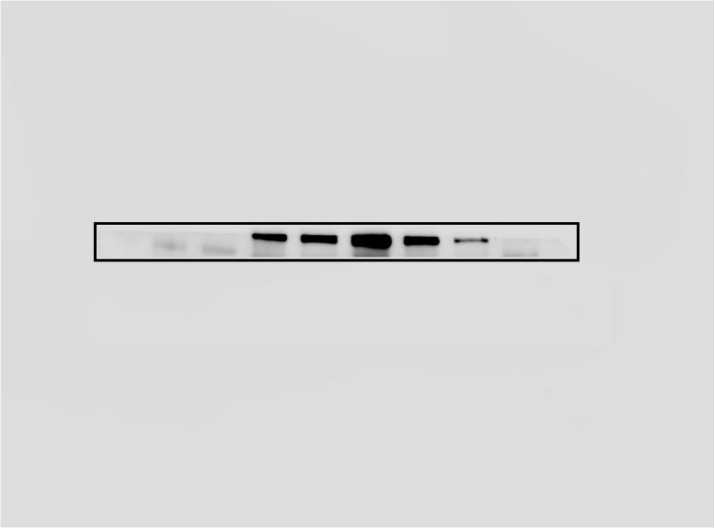

SCC-9-GAPDH C, M-NC, M, I-NC, I

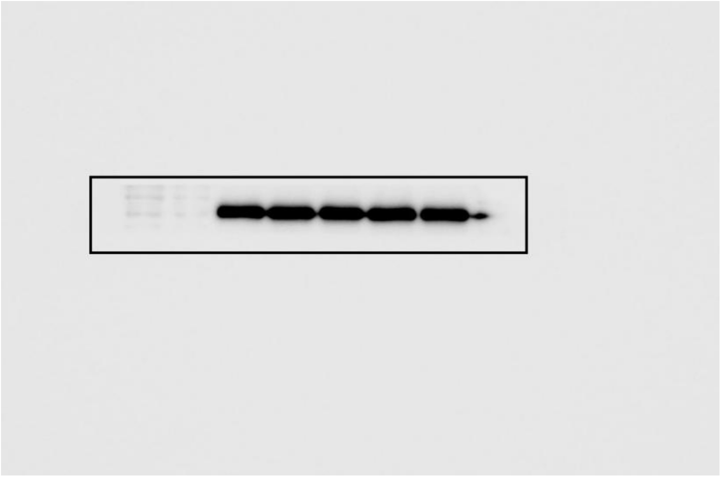

Fig5E: MiR-191 regulates the expression of β-catenin and the downstream genes of the β-catenin signaling pathway through targeting PLCD1. blank control (C)， miR-191 inhibitor+ siRNA-NC(I+si-NC), miR-191 inhibitor+siRNA-PLCD1(I+si-PLCD1)

CAL-27-β-catenin C I+si-NC I+si-PLCD1

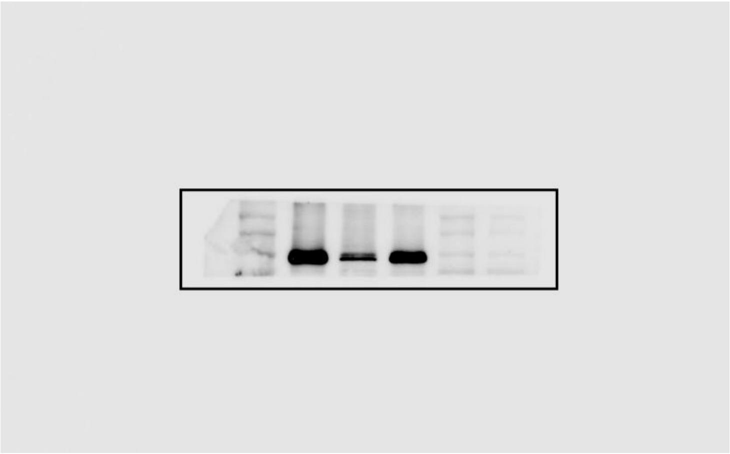

CAL-27-MMP9 C I+si-NC I+si-PLCD1

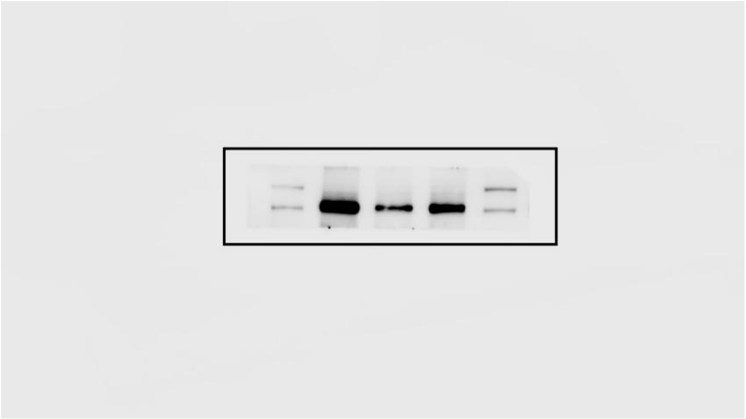

CAL-27-C-myc C I+si-NC I+si-PLCD1

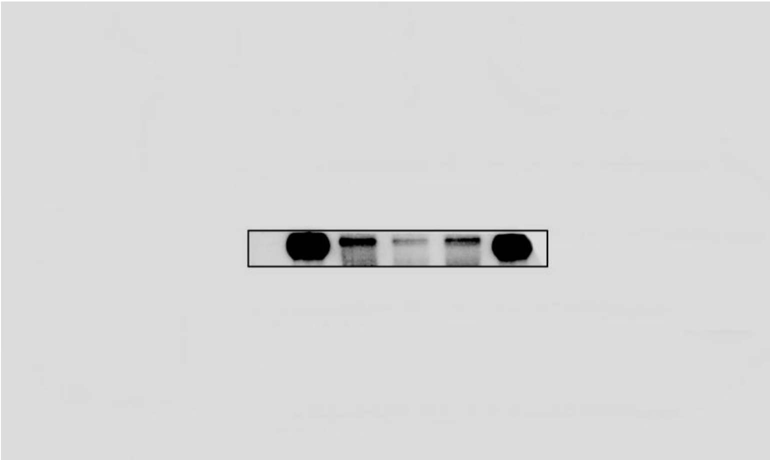

CAL-27-N-cadherin C I+si-NC I+si-PLCD1

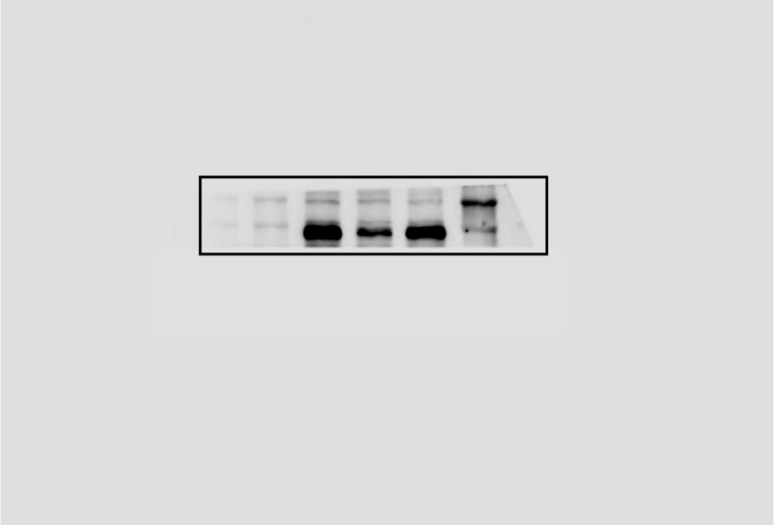

CAL-27- CDK4 C I+si-NC I+si-PLCD1

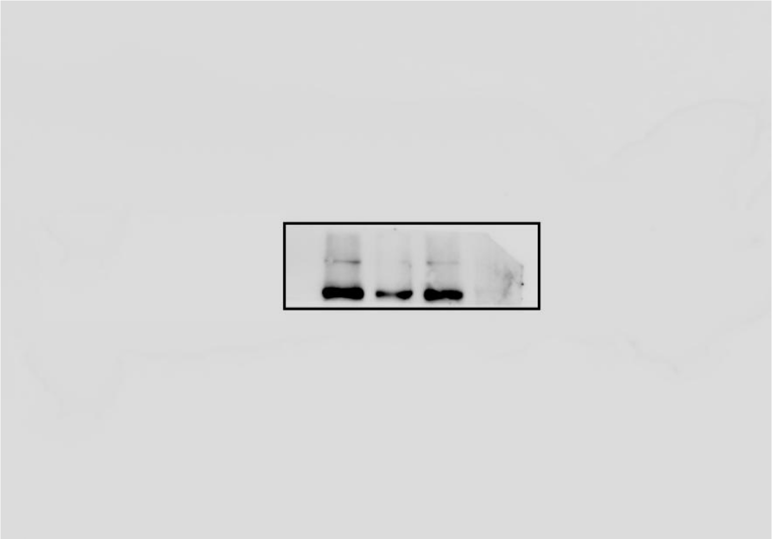

CAL-27- PCNA C I+si-NC I+si-PLCD1

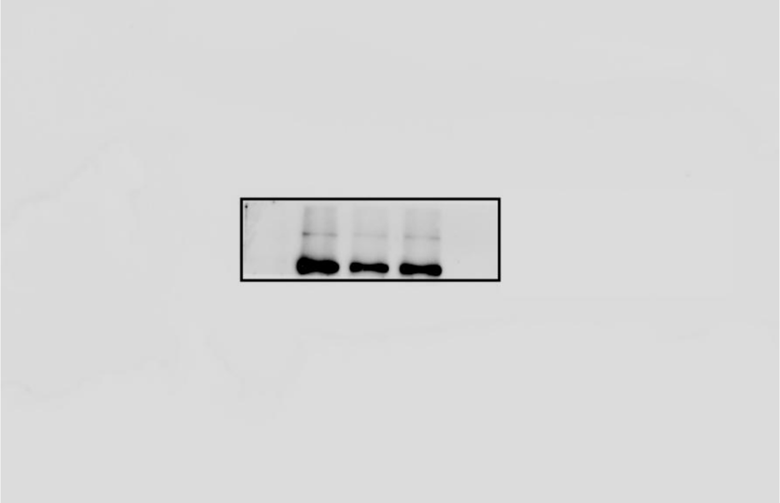

CAL-27-GAPDH C I+si-NC I+si-PLCD1

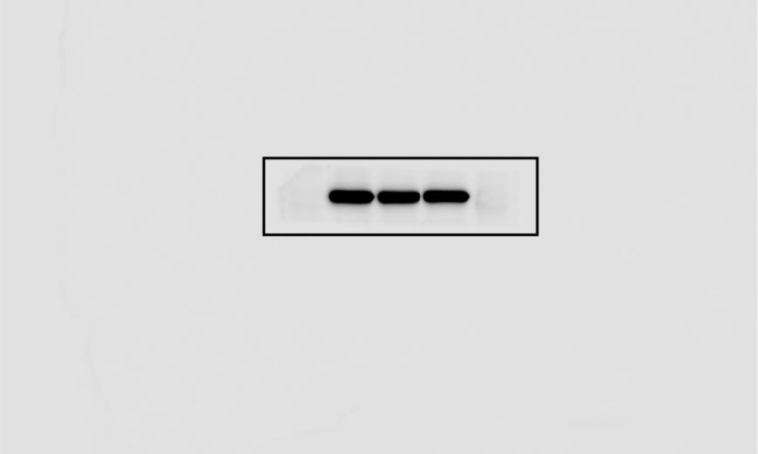

Fig5F: MiR-191 regulates the expression of β-catenin and the downstream genes of the β-catenin signaling pathway through targeting PLCD1. blank control (C)， miR-191 inhibitor+si-NC (I+si-NC), miR-191 inhibitor+si-PLCD1(I+si-PLCD1)

SCC-9-β-catenin C I+si-NC I+si-PLCD1

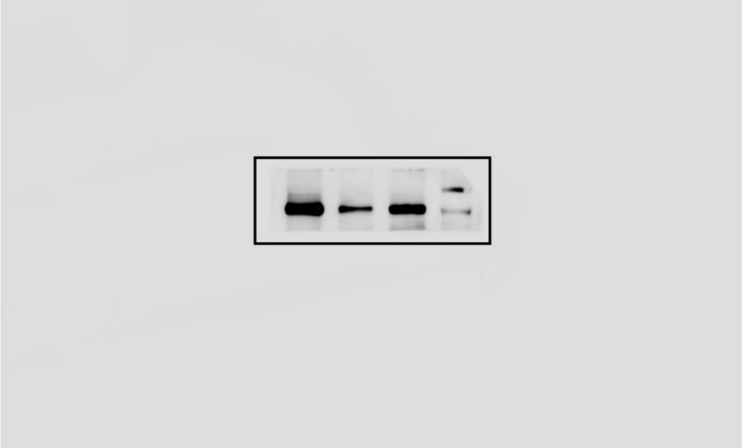

SCC-9-MMP9 C I+si-NC I+si-PLCD1

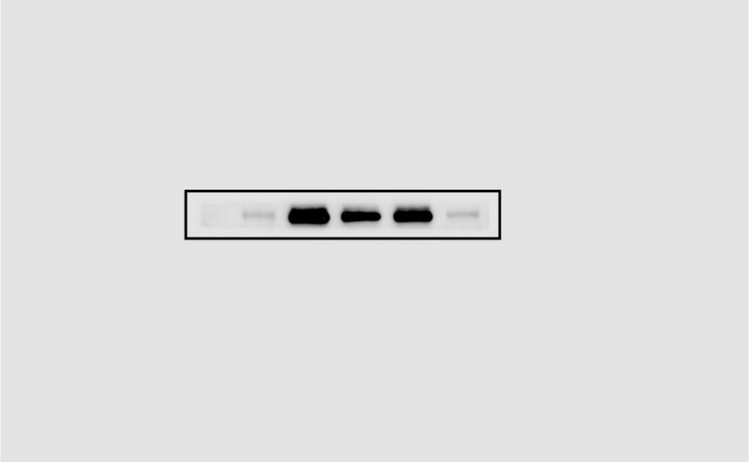

SCC-9-C-myc C I+si-NC I+si-PLCD1

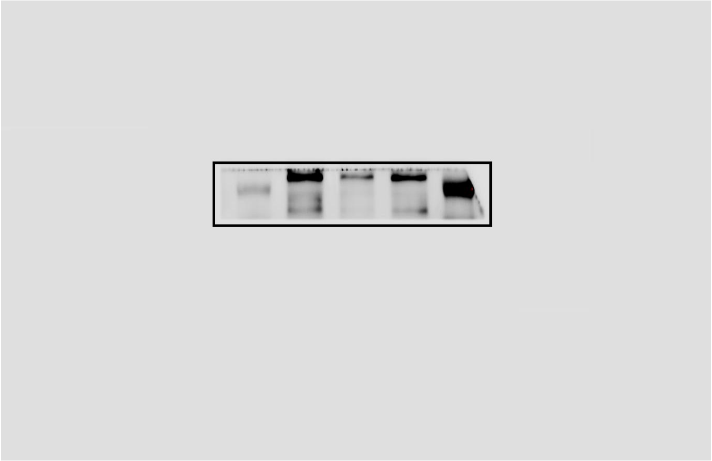

SCC-9-N-cadherin C I+si-NC I+si-PLCD1

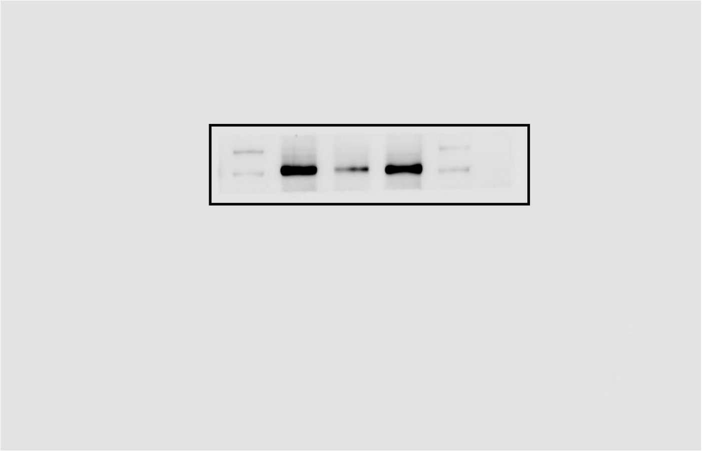

SCC-9-CDK4 C I+si-NC I+si-PLCD1

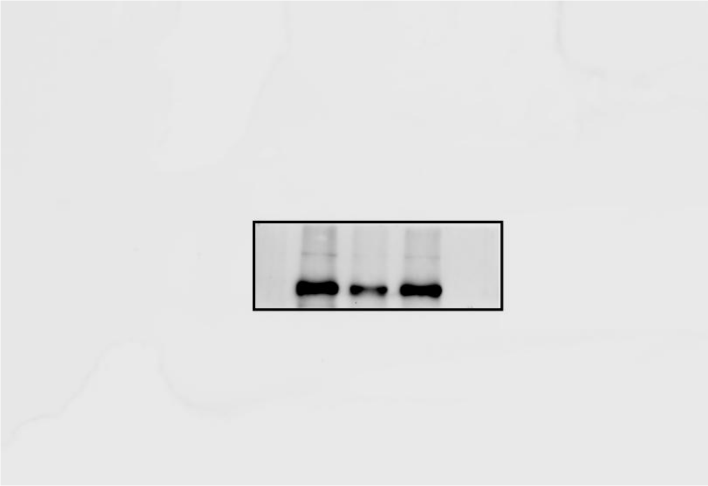

SCC-9-PCNA C I+si-NC I+si-PLCD1

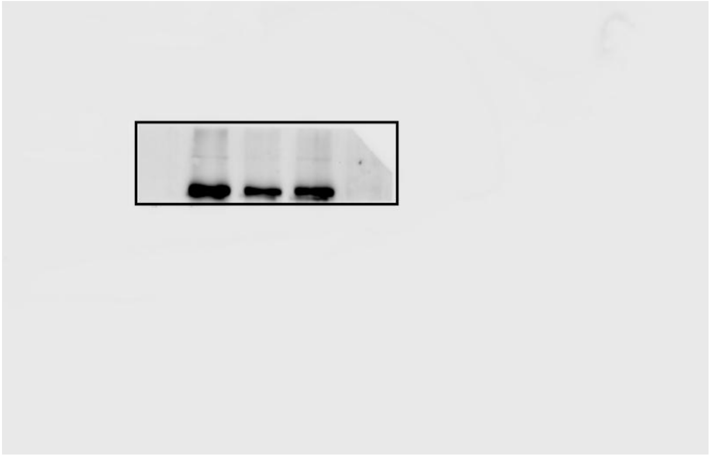

SCC-9-GAPDH C I+si-NC I+si-PLCD1

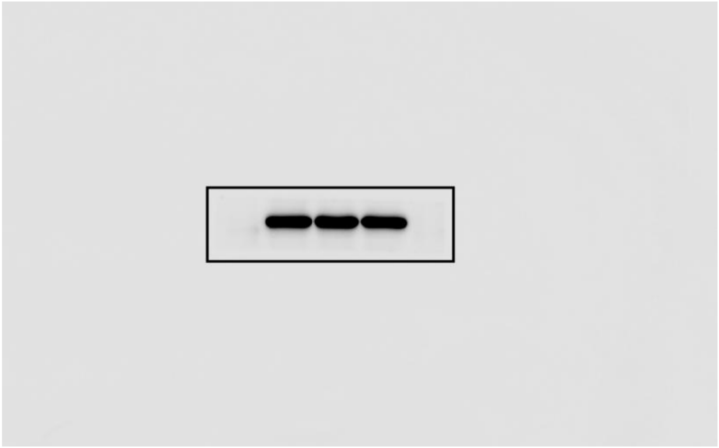

Supplement: Supplementary file 1 — Additional file 1. [file 12885_2023_11113_MOESM1_ESM.pdf]
